# Emerging roles of DYRK2 in cancer

**DOI:** 10.1074/jbc.REV120.015217

**Published:** 2021-01-07

**Authors:** Vasudha Tandon, Laureano de la Vega, Sourav Banerjee

**Affiliations:** Division of Cellular Medicine, School of Medicine, University of Dundee, Dundee, United Kingdom

**Keywords:** protein kinase, kinase inhibitor, proteasome, stress, phosphorylation, E3 ligase, proteostasis, CMGC, Cyclin-dependent kinases, Mitogen-activated protein kinases, Glycogen synthase kinases, and CDC-like kinases, CML, chronic myeloid leukemia, DYRK, Dual-specificity tYrosine phosphorylation–Regulated Kinase, EMT, epithelial–mesenchymal transition, GEMMs, genetically engineered mouse models, HIPK2, homeodomain-interacting protein kinase, HSF1, heat-shock factor 1, IFN, interferon, MM, multiple myeloma, NAPA, N-terminal autophosphorylation accessory, NOTCH1, neurogenic locus notch homolog protein 1, NSCLC, non–small-cell lung cancer, PEST, Pro-Glu-Ser-Thr, Ser62, serine62, Ser727, serine727, STAT3, signal transducer and activator of transcription 3, TBK1, TANK-binding kinase 1, TCGA, The Cancer Genome Atlas, Thr58, Threonine58, TNBC, triple-negative breast cancer

## Abstract

Over the last decade, the CMGC kinase DYRK2 has been reported as a tumor suppressor across various cancers triggering major antitumor and proapoptotic signals in breast, colon, liver, ovary, brain, and lung cancers, with lower DYRK2 expression correlated with poorer prognosis in patients. Contrary to this, various medicinal chemistry studies reported robust antiproliferative properties of DYRK2 inhibitors, whereas unbiased ‘omics’ and genome-wide association study-based studies identified DYRK2 as a highly overexpressed kinase in various patient tumor samples. A major paradigm shift occurred in the last 4 years when DYRK2 was found to regulate proteostasis in cancer *via* a two-pronged mechanism. DYRK2 phosphorylated and activated the 26S proteasome to enhance degradation of misfolded/tumor-suppressor proteins while also promoting the nuclear stability and transcriptional activity of its substrate, heat-shock factor 1 triggering protein folding. Together, DYRK2 regulates proteostasis and promotes protumorigenic survival for specific cancers. Indeed, potent and selective small-molecule inhibitors of DYRK2 exhibit *in vitro* and *in vivo* anti-tumor activity in triple-negative breast cancer and myeloma models. However, with conflicting and contradictory reports across different cancers, the overarching role of DYRK2 remains enigmatic. Specific cancer (sub)types coupled to spatiotemporal interactions with substrates could decide the procancer or anticancer role of DYRK2. The current review aims to provide a balanced and critical appreciation of the literature to date, highlighting top substrates such as p53, c-Myc, c-Jun, heat-shock factor 1, proteasome, or NOTCH1, to discuss DYRK2 inhibitors available to the scientific community and to shed light on this duality of protumorigenic and antitumorigenic roles of DYRK2.

Protein kinase DYRK2 is a member of the Dual-specificity tYrosine phosphorylation–Regulated Kinase (DYRK) family, which in turn belongs to the Cyclin-dependent kinases, Mitogen-activated protein kinases, Glycogen synthase kinases, and CDC-like kinases (CMGC) superfamily within the kinase complement of the human genome (1). The DYRK family consists of 5 members divided into two classes: Class I is comprised of DYRK1A and DYRK1B, whereas class II is comprised of DYRK2, DYRK3, and DYRK4 (Fig. 1*A*). DYRK2 is a class II DYRK that exhibits various structural features such as the NAPA or N-terminal autophosphorylation accessory domains (yellow/orange), DYRK-homology domain (green), activation loop segment (purple), nuclear localization sequence (red), the CMGC family–specific insert domain (gray) (Fig. 1*B*) most of which are conserved across the DYRK family (2). In DYRK2, specific loss-of-function mutations have been reported in cancer (Fig. 1*B*), which affect either the activity of the kinase or impede its ability to form functional complexes with interactors (3). In fact, phosphoproteomics studies show that these cancer mutations significantly alter substrate specificity of DYRK2 in cells (3). Class I DYRKs exhibit two distinct nuclear localization sequences and a stretch of polyserine and polyproline (PEST, Pro-Glu-Ser-Thr) domain with no distinct NAPA domains as in class II paralogues (Fig. 1*C*). Despite subtle structural differences between class I and II members, all DYRK isoforms exhibit a highly conserved autophosphorylation-mediated activation mechanism (4). During translation, hydroxylation of a highly conserved proline residue (proline441 for hDYRK2) on the inert/nascent kinase domain of DYRKs triggers a tyrosine autophosphorylation event within the activation loop (tyrosine382 for hDYRK2), which leads to conversion of the inactive to the active conformation of the kinase (5). In the fully active form, the DYRK transition from a tyrosine-phosphorylating kinase to a serine-/threonine-directed kinase, thus acquiring the label ‘dual specificity’ (2, 4). The NAPA and DYRK-homology domain domains are thought to promote the structural integrity of the nascent kinase enough to execute the indispensable autophosphorylation event (2). The CMGC-specific insert is conserved across the CMGC kinase superfamily and is proposed to play important roles in stabilization of the tertiary structure of the kinase and promoting complex formation with interactors/substrates (6).

**Figure 1:**
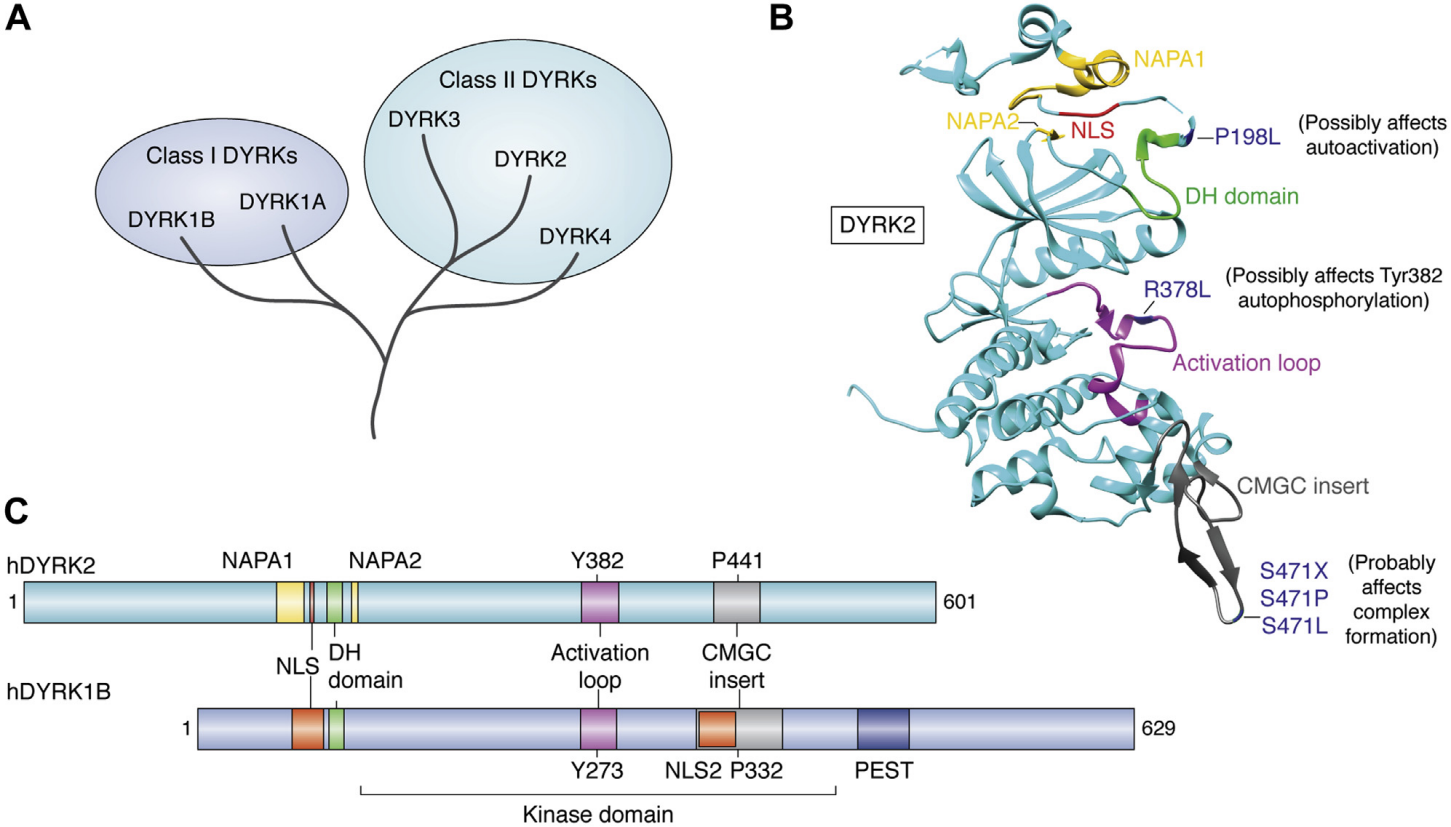
**DYRK2 belongs to the DYRK family within the CMGC superfamily and is mutated in cancer.***A*, DYRK2 is a class II DYRK on the CMGC superfamily branch of the kinome. *B*, structure of DYRK2 indicating the major structural domains and cancer-associated mutations (derived from PDB ID: 3K2L) with a hypothetical effect on DYRK2 structure/function. *C*, the domain diagram providing a 2D comparative image of the domains of class II DYRK2 and class I DYRK1B. Class I DYRKs exhibit two NLS sequences, a C-terminal PEST domain and a lack of NAPA domain characteristic of class II. The autophosphorylation of Tyr and hydroxylated Pro Y382/P441 (DYRK2) and Y273/P332 (DYRK1B) are shown. CMGC, Cyclin-dependent kinases, Mitogen-activated protein kinases, Glycogen synthase kinases, and CDC-like kinases; DYRK, Dual-specificity tYrosine phosphorylation–Regulated Kinase; NAPA, N-terminal autophosphorylation accessory; NLS, nuclear localization sequence; PEST, Pro-Glu-Ser-Thr.

Like most CMGC kinases, DYRKs have an amino acid motif of preference on their substrates. DYRKs prefer an arginine (R) at the −3 position of the phosphoserine/threonine residue along with a strong preference for a proline (P) at +1: Rxx(pS/T)P motif (7). Being a preferred motif across all members, redundancies have been observed wherein multiple CMGC kinases phosphorylate the same site on the substrate (reviewed in Boni *et al.* [8]). Although both the −3 R and +1 P are strongly preferred, some DYRK substrates lack the +1 P such as histone H3 for DYRK1A (9), 26S proteasome regulatory subunit 6B RPT3 (10), and heat-shock factor 1 (HSF1) (11) for DYRK2, whereas the −3 R is lacking on multiple DYRK2 substrates such as p53 (12, 13), c-Jun, c-Myc (14), and SIAH2 (15). For those lacking the +1 P, the substrates have exhibited no redundant kinases within the related CMGC superfamily thus far (10). Among the DYRKs, DYRK2 often functions in tandem with related CMGC kinase, GSK3, in sequentially phosphorylating various substrates (7, 9, 16, 17). DYRK2 provides a priming phosphorylation for further GSK3 activity (7, 9, 16, 17). DYRK2 has been identified in all eukaryotes (10, 18), and interestingly across all orthologues, the conserved biological function of the DYRK2 isoform is regulation of cell division and/or tissue development (10, 18). A recent work has shown that DYRK2 is an essential kinase during embryogenesis, and mouse embryos with homozygous deletion of DYRK2 exhibit stunted development and pups die just before birth (19).

Of all the DYRK isoforms, DYRK2 is the only member that functions as a kinase activity–independent scaffold for an E3 ubiquitin ligase complex (20, 21, 22, 23, 24). DYRK2 is an integral part of the EDVP (EDD [ubiquitin protein ligase] + DDB1 [damage-specific DNA-binding protein] + VPRBP [HIV-1 Vpr-binding protein]) E3 ubiquitin ligase complex that carries out phosphorylation-mediated degradation of various cell cycle components to ensure smooth transition of G2/M stages of cell cycle (20, 21, 22, 23, 24). Some of the cancer mutations in Figure 1*B* are thought to affect efficient EDVP complex formation (3). Thus, over the past few decades, many groups have identified various molecular mechanism and substrates for DYRK2 playing diverse roles in cellular growth, proliferation, and developmental processes with a focal point being its role in cancer (10, 25, 26, 27, 28).

Besides DYRK2, the other DYRK isoforms, especially the class I's, have a long history in the field of cancer. Although DYRK1B has an overall protumorigenic role specifically in pancreatic and ovarian cancers, DYRK1A exhibits a more controversial role with reports of both protumorigenic and antitumorigenic mechanism in different cancers (reviewed in Boni *et al.* [8]). Within the class II DYRKs, very little is known about DYRK3 and DYRK4 with limited literature pointing to a more protumorigenic role for both (29, 30). DYRK2, on the other hand, is the most extensively studied class II isoform, and the high-profile substrates reported, such as p53 (12, 13), c-Jun (14), c-Myc (14), NOTCH1 (31), HSF1 (11), 26S proteasome (10, 25, 26), and SIAH2 (15, 31), have brought the kinase to the forefront of oncology research. For the past 2 decades, multiple studies have reported an overarching tumor suppressor role of DYRK2 across various cancers (reviewed in Yoshida and Yoshida [27]), with antitumorigenic roles including regulation of cell cycle, apoptosis, epithelial–mesenchymal transition (EMT), cancer stemness, and antimetastatic roles (reviewed in Yoshida and Yoshida [27]). On the other hand, since 2016, multiple studies report major protumorigenic roles of DYRK2 (10, 11, 25, 26), and a few studies have identified DYRK2 as a possible cancer driver (32, 33, 34). Furthermore, mRNA expression analyses from The Cancer Genome Atlas (TCGA) tumors along with matched normal controls reveal that the majority of cancers have higher median expression of DYRK2 than adjacent normal tissues (26), and a similar pattern has been shown for DYRK2 protein levels in some tumor types (11, 26). All of these data suggest that DYRK2 might be an excellent potential drug target. With such high profile, oncology-related substrates, could the function of DYRK2 differ based on cancer type or cell type? To shed some light onto this question, this review will re-examine the current literature on the role of DYRK2 in cancer and follow up with existing knowledge of small-molecule inhibitors developed to target DYRK2.

## DYRK2 regulates proteostasis: an oncogenic role

**Figure 2:**
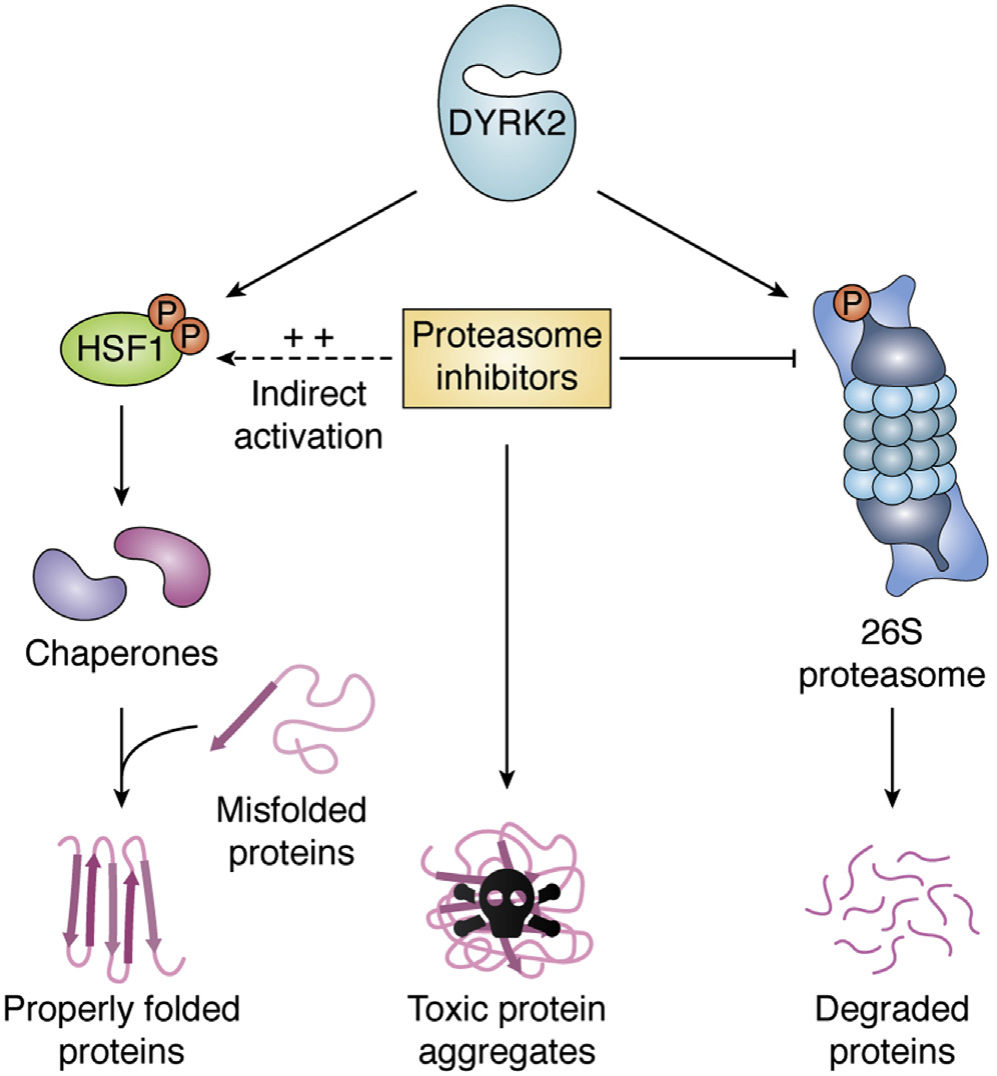
**DYRK2 regulates proteostasis *via* a two-pronged mechanism.** DYRK2 phosphorylates and upregulates the activity of the 26S proteasome, which reduces proteotoxic stress by degrading misfolded/unfolded proteins. In parallel, DYRK2 triggers phosphorylation-mediated activation of HSF1, which promotes transcriptional upregulation of chaperones that promotes folding of misfolded/unfolded proteins. Proteasome inhibitors (PIs) such as bortezomib, carfilzomib, and ixazomib inhibit the proteasome and result in enhanced proteotoxic stress because of toxic protein aggregates. Proteasome inhibition by PIs triggers indirect activation of the HSF1 pathway to compensate for the loss of proteasome activity thereby decoupling the proteasome dependence of cancer. HSF1, heat-shock factor 1.

DYRK2 maintains proteostasis of cancer cells by regulating two major players of the proteotoxic response pathway, which promotes the proper folding and/or degradation of proteins (Fig. 2). More than 90% of all solid human tumors carry numerous aberrations in chromosomes, referred to as aneuploidy (35). As a result of their severe aneuploidy, cancer cells are exposed to proteotoxic stress that increases the amount of toxic, unfolded proteins in the cell (36, 37). To survive proteotoxic stress, cancer cells can either increase protein folding capacity (controlled by the transcription factor HSF1) or increase the degradation of the misfolded/aggregated proteins (*via* the 26S proteasome and/or autophagy). DYRK2 phosphorylates and activates both HSF1 and the 26S proteasome and thereby activates the proteotoxic stress pathway promoting tumorigenesis in cancers such as triple-negative breast cancer (TNBC) and multiple myeloma (MM).

### DYRK2 regulates 26S proteasome function

In 2016, an RNAi kinase screen identified DYRK2 as a kinase-regulating 26S proteasome activity (10). The study showed that DYRK2 depletion either by si/shRNA or CRISPR/Cas9 KO led to a 30 to 40% decrease in proteasome activity (10). The mature 26S proteasome is a complex of more than 30 distinct subunits that catalyzes 80% of eukaryotic protein degradation and harbors three distinct peptidase activities in the core subunit (chymotryptic, tryptic, and caspase-like) (38, 39). Besides the core of the proteasome, the complex also consists of the 19S regulatory subunit that binds to ubiquitylated proteins, whereas a six-membered ATPase ring hydrolyzes the protein into a polypeptide chain for entry into the peptidase core for degradation (40). Interestingly, DYRK2 phosphorylates the Rpt3 subunit on the ATPase ring of the 19S subunit of the proteasome on an evolutionarily conserved Thr25 site (10). Rpt3 pT25 had been previously reported by Steve Gygi's group in their 2008 work on quantitative phosphoproteomics of mitosis (41), but the function of the phosphorylation was not known. A phospho-specific antibody generated against pT25 Rpt3 showed that the site is dynamically upregulated during G2/M stage of the cell cycle and that serum starvation leads to loss of Thr25 phosphorylation (10). Furthermore, CRISPR/Cas9 knock-in of a phospho-deficient Thr25Ala on Rpt3 mimics the DYRK2 KO phenotypes in cells wherein there is a delay in mitotic progression, slower cell proliferation rates, and inhibition of all three peptidase activities of the 26S proteasome (10). The 26S proteasome degrades nearly 80% of all eukaryotic proteins, and hence, a 30% loss in activity leads to significant proteotoxic stress and consequent cell death in breast cancer cells (10). Intriguingly, DYRK2 KO cells were significantly more sensitive to the proteasome inhibitor, bortezomib, suggesting DYRK2 could be a possible therapeutic target for treatment of cancer (10). Indeed, in an ectopic nude mouse xenograft model, DYRK2 KO and T25A Rpt3 knock-in cells were less efficient in generating a tumor as compared to parental cells (10). This study further established the DYRK2-proteasome axis as potentially tumor promoting because higher expression of DYRK2 significantly correlated with higher mortality and poorer relapse-free survival in patients with breast cancer (10). In fact, inhibition or genetic depletion of DYRK2 tipped the scales of proteostasis in TNBC and MM cells. DYRK2 mRNA levels are higher in newly diagnosed and relapsed MM than normal donors (26). In fact, mice bearing syngrafted/xenografted myeloma cells with genetic depletion of DYRK2 exhibit significantly slower myeloma disease progression and reduced bone degeneration (26). Furthermore, bortezomib-resistant RPMI8226 myeloma cells express higher protein levels of DYRK2 than nonresistant RPMI8226 (26), suggesting that DYRK2 might play a role in driving drug resistance in some myeloma cases. The potent and selective DYRK2 inhibitor, LDN192960, induces cytotoxicity in myeloma cells both *in vitro* and *in vivo* with minimal off-target effects (26). The fact that the DYRK2 inhibitor alleviates myeloma burden *in vivo* suggests DYRK2 could indeed be a viable *in vivo* target for myeloma therapeutics. Resistance to proteasome inhibitors have been reported in patients, and this is either brought about by cancer mutations in the proteasome core or *via* upregulation of HSF1-mediated proteotoxic response pathway.

### DYRK2 phosphorylates HSF1 and modulates proteotoxic response

The transcription factor HSF1 is the master regulator of proteotoxic stress responses and supports oncogenesis by helping cancer cells cope with the proteotoxic stress associated with both aneuploidy and oncogenic mutations. This has been demonstrated by the reduced susceptibility of *Hsf1*-KO mice to tumor formation driven either by *Ras*/*p53* mutations or by chemical carcinogens (42, 43). Furthermore, high levels of HSF1 expression associate with poor outcome of various cancers (44). Upon proteotoxic stress, HSF1 is activated, translocates to the nucleus (45), and initiates the transcription of heat-shock proteins. Heat-shock proteins then function as molecular chaperones, protecting cells against proteotoxic stress by assisting in protein folding (46). HSF1 activity and stability are tightly controlled by multiple post-translational modifications (47). Among these, phosphorylation of serine 320 and serine 326 is associated with stability and nuclear accumulation followed by enhanced transcriptional activity of HSF1 (48, 49, 50). DYRK2 positively regulates HSF1 nuclear stability and activity, by phosphorylating it at Ser320 and Ser326 in TNBC cells (11). Indeed, DYRK2-depleted TNBC cells were far more sensitive to heat shock–mediated proteotoxic stress than parental cells, thus corroborating that DYRK2 plays a major role in maintaining proteostasis in TNBC cells. This link between DYRK2 and HSF1 is also observed in TNBC tumor samples, wherein a marked correlation was observed between high DYRK2 levels and high nuclear HSF1 levels.

The HSF1 pathway and the proteasome are not just two of the main pathways maintaining cell proteostasis, but they are interconnected and can compensate for each other. As mentioned before, proteasome inhibitors lead to the activation of HSF1 (Fig. 2) in an effort to protect the cell against the accumulation of toxic proteins (51, 52). The cytoprotective response mediated by HSF1 counteracts the cytotoxic effect of proteasome inhibitors (51, 52, 53), and thus, HSF1 inhibition might be effective to overcome proteasome inhibitor resistance in cancer cells. In that sense, a DYRK2 inhibitor induced cytotoxicity even in MM cells resistant to proteasome inhibitors (25, 26), suggesting that in fact DYRK2 inhibition might be targeting different complementary pathways. This observation was further echoed by a recent study showing that MM cells were extremely sensitive to increased temperatures and heat shock (54). In fact, combining heat shock with proteasome inhibitors led to higher accumulation of misfolded proteins leading to acute proteotoxic stress and apoptosis in the myeloma cells (54). Because cancer cells harbor significantly higher misfolded proteins than normal cells, targeting DYRK2 could indeed tip the scales for proteostasis in malignant cells and provide a significant therapeutic window for targeting specific cancers. This is indeed the case because normal/noncancerous cells were far more resistant to DYRK2 inhibitors (25, 26). Thus, targeting DYRK2 can significantly affect proteostasis (Fig. 2) *via* perturbation of both HSF1 and 26S proteasome activity leading to cancer cell death.

Hence, in the context of TNBC and MM, DYRK2 plays an overarching role as an oncogenic kinase and a potential therapeutic target.

## DYRK2-p53 tumor suppressor link

A major molecular mechanism by which DYRK2 has been reported to exhibit the antitumorigenic role is *via* phosphorylation of tumor suppressor p53 on serine46 (Ser46). Upon genotoxic stress, energy stress, or heat shock, multiple CMGC kinases such as homeodomain-interacting protein kinase (HIPK2), mitogen-activated protein kinase p38α, and DYRK2 can phosphorylate p53 on Ser46, which triggers transcription of proapoptotic genes leading to cell death or cell senescence (reviewed in Liebl and Hofmann [55]). Upon DNA damage, DYRK2 is phosphorylated by ataxia-telangiectasia mutated kinase, which protects DYRK2 from proteasomal degradation leading to its nuclear accumulation where it phosphorylates p53 on Ser46 and promotes its transcriptional tumor suppressor activity (12, 13). Although phosphorylated Ser46 on p53 is indeed a marker for its tumor suppressor role, DYRK2 by no means is the exclusive kinase here. With multiple kinases including PKCδ, HIPK2, ataxia-telangiectasia mutated kinase, and p38α phosphorylating Ser46 upon genotoxic stress (55), it is hard to decipher to what extent DYRK2 contributes to this tumor suppressor role. Furthermore, p53 is mutated or truncated in a vast number of solid tumors and cancer patients with altered p53 exhibit significantly poorer survival (56, 57). Mutated p53 often exhibits stoichiometrically lower phosphoSer46 (58) and has been reported to trigger pro-oncogenic functions upon phosphorylation (59). This suggests that p53 phosphorylated on Ser46 serves as a tumor suppressor only in the few percentage of cancers containing WT p53 where patients exhibit better chances of survival.

Multiple publications carrying out sequencing or immunohistochemistry to study mRNA/protein levels of DYRK2 have suggested that DYRK2 is a tumor suppressor in colorectal (60, 61, 62, 63), liver (64), brain (65), and lung cancers (66, 67) and that the kinase promotes chemosensitivity in ovarian cancer (68). However, ovarian, liver, brain, lung, and colorectal cancers exhibit some of the highest mutations and variant allele frequencies in p53 compared with other cancer types (56, 57, 69). Thus, it is unclear to what extent DYRK2's phosphorylation of p53 could play as a tumor-suppressive role in these solid tumors exhibiting p53 mutation or loss. Furthermore, in endothelial cells, the pan-DYRK inhibitor, harmine (albeit with possible off-target effects), promotes p53 phosphorylation on Ser15, Ser20, and Ser37 (70), leading to higher p53 protein levels upon DNA damage (70, 71). Seemingly, in this case, DYRK2 inhibition led to tumor suppression. Hence, it is also important to decipher the molecular functions of DYRK2 in noncancer models or as a potential cancer driver. A recent unbiased deep multiomics study looking at the proteome, phosphoproteome, and transcriptome of murine high-grade brain cancer glioma model reported 41 kinases including DYRK2 exhibiting higher activity and rewired substrate signaling (34). Furthermore, the glioma murine model was generated by intracranial implantation of genetically engineered p53 null astrocytes, thus making the tumor-suppressor role of DYRK2-p53 axis highly untenable in this model.

**Figure 3:**
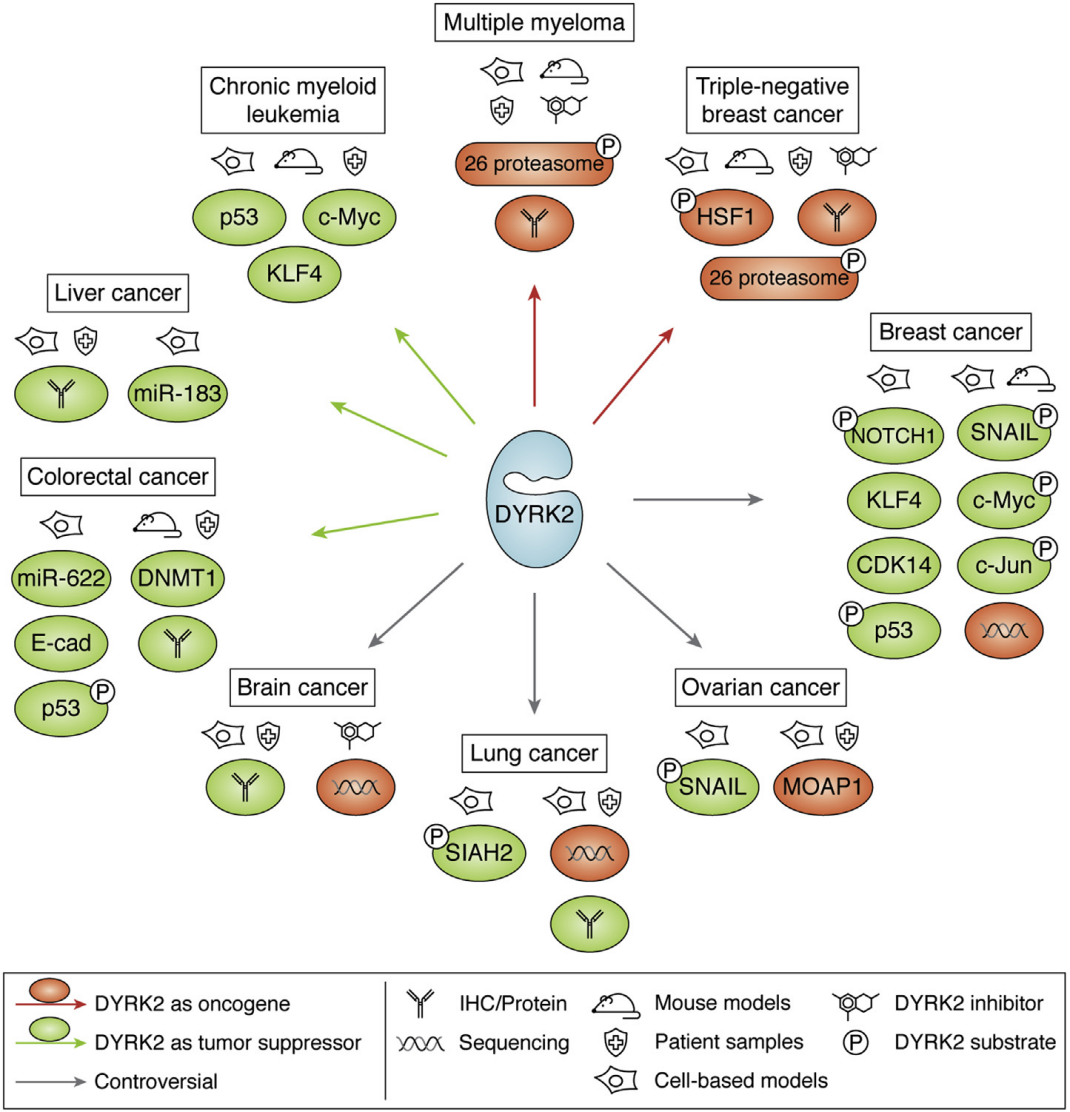
**Overall summary of DYRK2 in neoplasia.** The figure provides a holistic view of the various reported roles of DYRK2 in different forms of cancers. For each cancer, the various interactors/substrates/effectors of DYRK2 are highlighted either in *green* (tumor-suppressor role) or in *red* (protumorigenic role). The cancer models/tools (cell-based, mouse models, patient samples, DYRK2 inhibitor) used to derive the respective conclusions are also shown. Direct DYRK2 substrates are shown with the added (P) phosphate, and conclusions based on sequencing or immunohistochemistry are also highlighted. *Gray arrows* indicate those cancers where controversial or conflicting reports have been documented.

Besides solid tumors, chronic myeloid leukemia (CML) cell lines exhibit significantly lower protein levels of DYRK2 than other hematological cancer cell lines (72). Interestingly, transcriptional upregulation of DYRK2 inhibits survival and self-renewal of CML stem/progenitor cells *via* c-Myc depletion and p53 activation (72). This tumor-suppressor role of DYRK2 seems to be CML specific because all other leukemia subtypes tested exhibited naturally elevated protein levels of DYRK2 at basal conditions (72), suggesting alternate driving mechanisms for tumorigenesis. Hematological malignancies exhibit fewer p53 mutations/loss (56, 57), and hence, DYRK2 could indeed be a tumor suppressor in specific subtypes such as CML. On a similar note, silencing DYRK2 has been reported to increase cell proliferation and reverse cell adhesion–mediated drug resistance in non-Hodgkin's lymphoma cell lines (73). Intriguingly, MM cells are highly sensitive to DYRK2 inhibition irrespective of p53 status (25, 26). Because DYRK2 inhibition in myeloma tips the balance of proteotoxic stress (26, 54), all cells whether p53 WT (such as cell line MM.1S) or mutated (such as cell lines RPMI8226 and U266B1) die (26). This suggests that in myeloma, the role of DYRK2 as an oncogenic driver probably plays a far greater role than its tumor-suppressor function potentiated by p53 phosphorylation. Hence, stratification of cancer subtypes before assigning molecular functions to DYRK2 is important. However, DYRK2 has tumor-suppressor mechanisms beyond p53 involvement, and it is important to investigate the diverse mechanisms at play to derive a larger perspective (Fig. 3).

## Other molecular mechanisms linking DYRK2 and cancer

Various p53-independent tumor-suppressor mechanisms have been reported for DYRK2, while other substrates point to an oncogenic role. Each mechanism focuses on specific cancer types and subtypes (Fig. 3 and Table 1). The main substrates and mechanisms are critically presented below.

**Table 1:** DYRK2 molecular mechanism and substrates/partners listed along with reported phosphorylation sites, overarching role, and the unanswered questions raised by each study

Cancer	Molecular partner	Phosphosite	Reported role	Pending question/issue
Breast	26S proteasome/RPT3 (10, 25, 26)	Thr25	Oncogenic; regulates proteostasis	Data specific for triple-negative breast cancer subtype.
	HSF1 (11)	Ser320, Ser326		Data specific for triple-negative breast cancer subtype; possible redundancy with other kinases.
	SNAIL (83)	Ser104	Tumor suppressor; EMT downregulation	Data heavily reliant on ectopic overexpression; possible redundancies with other kinases
	NOTCH1 (31)	Thr2512	Tumor suppressor; reduces invasion	Redundancies with other kinases; C-Myc pSer62 can be oncogenic; redundancies with other kinases; small sample sizes used for *in vivo* work.
	C-Myc (14)	Ser62		
	C-Jun (14)	Ser243		Possible redundancies with other DYRK family kinases
	p53 (12, 13)	Ser46	Tumor suppressor; proapoptotic upon genotoxic stress	Most tumors are p53 mutated/null and mutated p53 becomes oncogenic; DYRK2 mRNA strongly overexpressed in breast cancer overall.
	CDK14 (129)	n/a	Tumor suppressor; reduces invasion and proliferation	No specific mechanism reported on how DYRK2 regulates CDK14 transcription.
Lung	SIAH2 (86)	Ser16, Thr26, Ser28, Ser68, Thr119	Tumor suppressor; modulates hypoxia response pathways	DYRK2 is strongly overexpressed in lung adenocarcinoma; redundancies with other kinases
Ovarian	With EDVP/EDD only (89)	n/a	Oncogenic; degrades proapoptotic MOAP1; chemoresistance	Phosphorylated substrate (if any) not established.
	SNAIL (68)	Ser104	Tumor-suppressor; EMT downregulation; chemoresistance	Data heavily reliant on ectopic overexpression; possible redundancies with other CMGC kinases; 2 cell lines used only.
Brain	PI3K/AKT/GSK3β (65)	n/a	Tumor-suppressor; EMT downregulation	DYRK2 mRNA is strongly overexpressed in glioma; DYRK inhibitors kill glioma cells.
	Unknown (unbiased multiomics) (34)		Oncogenic; DYRK2 increased activity and rewired signaling	Multiomics data derived from p53 null murine glioma models; no direct mechanism studied.
Multiple myeloma	26S proteasome/RPT3 (25, 26)	Thr25	Oncogenic; regulates proteostasis	n/a
Leukemia	p53/c-myc/KLF4 (72)	n/a	Tumor suppressor; reduces cancer stemness	Data specific for chronic myeloid leukemia subtype.
Liver	p53/c-myc (62, 64, 130)	n/a	Tumor suppressor; EMT downregulation; reduces invasion; chemoresistance	p53 and c-Myc have extensive oncogenic mutations reported in liver cancer.
	LNC-HC/hsa-miR-183-5p (104)		Tumor suppressor; transcriptional upregulation of DYRK2	Multiple tumor suppressors upregulated including DYRK2; no specific DYRK2 mechanism reported.
Colorectal	p53 (12)	Ser46	Tumor suppressor; proapoptotic upon genotoxic stress	Most colorectal tumors are p53 mutated/null, and mutated p53 becomes oncogenic; DYRK inhibitor promotes p53 phosphorylation.
	DNMT1 (103)	n/a	Tumor suppressor; epigenetic downregulation of DYRK2	No specific DYRK2 mechanism reported.
	miR-622 (63)		Tumor suppressor; EMT downregulation; reduces invasion	
Lymphoma	CDK2/p27Kip1 (73)	n/a	Tumor suppressor; EMT downregulation; chemoresistance	Data specific for non-Hodgkin's lymphoma subtype.

CMGC, Cyclin-dependent kinases, Mitogen-activated protein kinases, Glycogen synthase kinases, and CDC-like kinases; DYRK, Dual-specificity tYrosine phosphorylation–Regulated Kinase; EMT, epithelial–mesenchymal transition; HSF1, heat-shock factor 1; MOAP, modulator of apoptosis protein 1; NOTCH1, neurogenic locus notch homolog protein 1; n/a, not directly reported.

Also, refer Figure 3.

### c-Myc

DYRK2 has been reported to exhibit a p53-independent tumor-suppressor role by phosphorylating c-Myc on serine62 (Ser62) (14). c-Myc is a major proto-oncogenic transcription factor known to be overexpressed and mutated in various cancers (74). Post-translational modifications of c-Myc have been a topic of much debate over the past 30 years in which sequential phosphorylation of Ser62 and Threonine58 (Thr58) seems to play major roles in c-Myc transactivation (75, 76). The consensus in the field is that Thr58 is a GSK3-phosphorylation site while Ser62 seems to be the priming site for GSK3 activity, and similar to phosphorylation of p53 at Ser46, various CMGC kinases have been proposed (75), including DYRK2 to phosphorylate c-Myc. Dual phosphorylation of c-Myc on Thr58 and Ser62 triggers binding to an E3 ubiquitin ligase SCF-Fbxw7 (Skp-Cullen-F-box) and consequent proteasomal degradation of c-Myc (14), thus leading to the proposed tumor suppressor role of DYRK2. As stated previously, transcriptional upregulation of DYRK2 in CML promotes c-Myc degradation (72). In Burkitt lymphoma, nearly 60% of patients exhibit mutation of the GSK3 site, Thr58 (77), whereas primary cells exhibit lower levels of Thr58 phosphorylation (76), thus suggesting a tumor-suppressor role of GSK3 in this context. However, increased Ser62 phosphorylation has been observed in immortalized cells compared with primary cells (76), and monophosphorylation of Ser62 has been linked to c-Myc stabilization and higher transcriptional activity in multiple studies (78, 79). This indicates a similar conundrum as observed with the p53 Ser46 site wherein multiple kinases and diverse cancer subtypes exhibit altered mechanisms of action of major cancer-associated genes such as p53 and c-Myc.

### c-Jun

A similar story is observed in case of c-Jun wherein two phosphorylation sites serine249 (Ser249: a bona fide GSK3 site) and Ser243 (reported to be phosphorylated by DYRK2) have been reported (14, 80). c-Jun is a transcription factor with established oncogenic roles (80). Similar to c-Myc, the E3 ubiquitin ligase SCF-Fbxw7 degrades c-Jun upon dual phosphorylation of Ser249 and Ser243 (81). Unlike c-Myc, Ser243 on c-Jun could be a DYRK-specific site because a previous study has elegantly ruled out most of the other CMGC kinase families (80). The same study did, however, observe redundancies between DYRK1A and DYRK2 for Ser243 on c-Jun *in vitro* (80), which is not surprising because the site is a +1P. In fact, dephosphorylation of Ser243 enhances c-Jun transcriptional activity in patients with cervical cancer exhibiting lower phosphoSer243 c-Jun in their tumors (82). Although the question of intra-DYRK redundancy remains, phosphoSer243 on c-Jun could indeed be a tumor-suppressor marker in specific cancers.

### SNAIL

DYRK2 has also been reported to phosphorylate the zinc finger domain containing protein SNAIL that plays essential roles during development by triggering EMT (68, 83). DYRK2 knockdown led to upregulation of mesenchymal markers with consequent downregulation of epithelial E-cadherin mRNA in colon cancer (61) and promotion of proliferation and migration of glioma cells (65). This observation was consistent with other studies reporting downregulation of DYRK2 in metastatic colorectal secondary tumors found in the liver (62). SNAIL has been reported to be overexpressed in specific cancers and promote oncogenic progression by promoting EMT, invasion, and metastasis (83). DYRK2 phosphorylates serine104 on SNAIL that provides a priming site for GSK3, triggering the phosphorylation-mediated degradation of SNAIL (68, 83). This mechanism of antitumorigenic activity by DYRK2 is thought to promote chemosensitivity for ovarian cancer cells (68). A follow-up study reports that the DYRK2-mediated degradation of SNAIL is in fact reversed by p38α kinase (84). Although an interesting molecular mechanism, in both studies, DYRK2 ectopic overexpression has been carried out to justify the phosphorylation. Overexpression of CMGC kinases often leads to nonphysiological false-positive subcellular localizations and substrate identifications because of redundancy and high affinity for +1 P sites and hence further tools need to be used to confirm the DYRK2–SNAIL mechanism.

### SIAH2

Seven In Absentia Homolog 2 or SIAH2 is an E3 ubiquitin ligase that plays a major role in targeted degradation of various proteins playing essential roles in regulating hypoxia (85). SIAH2 specifically regulates hypoxic tumor microenvironment by downregulation of key kinases in the Hippo signaling pathway (85). Furthermore, higher expression of SIAH2 is observed in lung cancer (86) and it plays oncogenic roles in castration-resistant prostate cancer (87). Interestingly, DYRK2 phosphorylates SIAH2 on 5 residues Ser16, Thr26, Ser28, Ser68, and Thr119. These modifications alter its subcellular localization thereby rewiring SIAH2 substrate specificity (15). SIAH2, in turn, is capable of degrading DYRK2 in specific cancers thereby triggering a protumorigenic hypoxic microenvironment (15, 85, 86). Some kinase redundancy has been observed wherein p38α kinase is capable of phosphorylating SIAH2 on same sites as DYRK2 (88); however, the DYRK2–SIAH2 link points to an interesting interplay between a kinase and a ubiquitin ligase regulating each other and thereby balancing protumorigenic and antitumorigenic roles.

### EDVP E3 ubiquitin ligase

As stated previously, DYRK2 forms a kinase-independent scaffold for the EDVP E3 ligase complex and a recent study has reported loss-of-function point mutations of DYRK2 in cancer, which largely alters the interactome and substrate specificity of DYRK2 (3). The recurrent mutations (Fig. 1*B*) are thought to alter activity and/or formation of the EDVP complex (3). As part of the EDVP complex, DYRK2 phosphorylates and triggers degradation of multiple substrates such as katanin p60 (KATNA1) (23), telomerase reverse transcriptase (TERT) (22), and centrosome protein 110 (CP110) (21). Phosphorylation-mediated degradation of these substrates are required for proper cell cycle transitions especially the G2/M stage. Cancer mutations could result in incomplete EDVP complex formation, and incomplete EDVP can exhibit oncogenic prosurvival role because DYRK2+EDD alone degrades the proapoptotic factor modulator of apoptosis protein 1 independently of DDB1 and VPRBP in ovarian cancer (89). The substrates of DYRK2–EDVP exhibit both protumorigenic and antitumorigenic roles in various cancers thus adding further complexity. Ovarian cancer patients with higher levels of KATNA1 exhibit better overall survival (90); higher CP110 can decrease breast cancer cell invasion (91), yet lung cancer tissue expresses higher CP110 than the normal lung (92), while TERT is largely oncogenic (93). Thus, DYRK2–EDVP functions are tumor specific.

### STAT3

DYRK2 has been reported to phosphorylate signal transducer and activator of transcription 3 (STAT3) *in vitro* (94). STAT3 is a transcription factor with both oncogenic and tumor-suppressor roles including regulation of tumor microenvironments (reviewed in Galoczova *et al.* [95]). STAT3 is phosphorylated on various residues upon interleukin/cytokine stimulation, and the phosphorylation on serine727 (Ser727) is thought to be an oncogenic biomarker in some subtypes of breast cancer (96). Although Ser727 is thought to promote the transcriptional activity of STAT3 (95), various kinases (CMGC family and beyond) have been reported to target Ser727 which is a +1P site (94). Thus, it is very difficult to dissect the importance of DYRK2 alone in driving phosphoSer727-mediated STAT3 activity.

### TBK1

TANK-binding kinase 1 (TBK1) is an important upstream regulator of innate immune transcription pathways triggering type I interferon (IFN) translation and signaling in response to pathogens (97). DYRK2 phosphorylates TBK1 at serine527, which leads to phosphorylation-mediated degradation of TBK1 and downregulation of type I IFN signaling upon viral infection (97). Besides infections, the elevated presence of type I IFN correlates with a favorable prognosis in patients with different cancers (98, 99). In fact, reduced IFN-related gene expression leads to an immunosuppressive tumor microenvironment resulting in immunotherapy resistance in many solid tumors (99). Thus, DYRK2-mediated downregulation of IFN signaling could play a major oncogenic role in triggering immunotherapy resistance in various cancers. However, the study reporting DYRK2 as the upstream kinase of TBK1 relies on ectopic overexpression of DYRK2 to demonstrate direct phosphorylation of a canonical +1P motif (97). There could be redundancies with other CMGC kinases at that site which needs to be addressed more thoroughly.

### NOTCH1

In response to chemotherapeutic agents, DYRK2 facilitates phosphorylation-mediated degradation of neurogenic locus notch homolog protein 1 (NOTCH1), which acts as an antiproliferative mechanism in breast cancer cells (31). NOTCH1 is a single transmembrane receptor and triggers intracellular signaling *via* binding to specific ligands (31). DYRK2 phosphorylates NOTCH1 on threonine2512 (Thr2512), which is a +1P site. However, NOTCH1 exhibits both tumor suppressor and oncogenic roles on a cancer-type basis (100). Interestingly, Thr2512 lies in the intracellular carboxy-terminal region of NOTCH1 that exhibits a PEST domain. The PEST region is the target of multiple CMGC kinases such as DYRK1A, HIPK2, CDKs, and GSK3, which triggers hyperphosphorylation and proteasomal degradation of NOTCH1 (reviewed in Lee *et al.* [101]). Thus, the redundancy conundrum remains to be solved to understand the function of NOTCH1's phosphorylation by DYRK2.

### Transcriptional/epigenetic mechanisms

Besides modulating substrate phosphorylations, transcriptional and epigenetic mechanisms of DYRK2 regulation have also been proposed for some cancers. Specifically, the downregulation of DYRK2's gene expression has been linked to increased stemness in breast cancer (102) and CML (72) *via* upregulation of transcription factor Krüppel-like factor 4. DYRK2 expression was also downregulated transcriptionally by DNA methyltransferase 1 in colon cancer (103). The DYRK2 promoter region exhibited a higher level of methylation in cancer tissues than healthy tissues while treatment of cells with hypomethylating drug 5-azacytidine increased DYRK2 mRNA and protein levels (103). Furthermore, DYRK2 was reported to downregulate oncogenic miR-622 expression and reverse invasion of cancer cells (63), whereas long noncoding RNA long noncoding RNA derived from hepatocytes inhibits the proliferation of liver cancer cells by rescuing the expression of DYRK2 (104).

To reiterate, multiple molecular mechanisms have been proposed for DYRK2, and each mechanism is cancer-type or subtype specific (Fig. 3 and Table 1). The controversial role of DYRK2 is best highlighted in breast and lung cancers.

## DYRK2 and breast cancer: a major controversy

### TNBC

Various studies have focused on the role of DYRK2 in TNBC (10, 11, 25, 26). These studies revealed that both mRNA and protein levels of DYRK2 were higher in TNBC tumors than adjacent normal breast tissues (26). Complementing this information, a recent study with 715 samples of patients with breast cancer have shown that high protein levels of nuclear DYRK2 were associated with significantly reduced cancer survival and a shorter time to recurrence specifically within the TNBC subtype cohort (11). To test the potential therapeutic value of targeting DYRK2 in TNBC, three studies have compared the ability of parental and DYRK2-deficient TNBC cell lines to produce tumors *in vivo* (10, 25, 26). Crispr/Cas9-mediated DYRK2 deletion in MDA-MB-231 or MDA-MB-468 cells showed that tumors derived from TNBC–DYRK2–deficient cells had significantly slower growth rates and lower tumor burden than those derived from their parental cells. Importantly, two studies have shown that treatment with the DYRK2 inhibitors, curcumin and LDN192960, impaired growth of established TNBC tumors (25, 26). In contrast with these findings, other studies have used MDA-MB-231–derived xenografts and reported DYRK2 control EMT by degrading SNAIL (83) and promoting transcription factor Krüppel-like factor 4 expression (102), thereby functioning as a tumor suppressor. Both the studies used a DYRK2 overexpression system to show that higher DYRK2 decreased tumor formation. One study reported that mice xenografted with DYRK2-overexpressing MDA-MB-231 cells showed few metastatic lesions and a prolonged survival compared with those injected with control cells (83). In a second study, the authors compared the number of tumors produced by injecting increasing numbers of MDA-MB-231 cells with or without overexpressed DYRK2 (102). The authors used a sample size of n = 6 mice per condition and show that the total number of tumors derived from DYRK2-overexpressing cells was marginally lower than controls (102). This is in sharp contrast to others reporting DYRK2 depletion reduces proliferation and tumor formation potential of MDA-MB-231 cells (10, 11, 25, 26, 105). Some of these discrepancies might be due to the differential approaches used (DYRK2 knockdown/KO *versus* overexpression systems) or due to underpowered sample sizes. Furthermore, a phosphotyrosine proteomics study in TNBC cells reported that DYRK2 was among the top 5 phosphorylated proteins observed in aggressive basal-like TNBC cells (105). Because there is no evidence of the activation loop tyrosine exhibiting altered stoichiometric phosphorylation, the high levels of phosphorylation observed could be due to higher DYRK2 protein levels. In fact, siRNA knockdown of DYRK2 in basal-like TNBC MDA-MB-231 and HCC1395 cells lead to reduced proliferation, invasion, and colony formation potential of the cells (105).

### Other breast cancer subtypes

Multiple studies looking at the role of DYRK2 in breast cancer have used the hormone receptor–positive and HER2-negative MCF7 cell line for xenograft studies. In the main study that supports the tumor-suppressor role of DYRK2 in breast cancer, the group identified DYRK2 as a priming kinase for c-Jun and c-Myc (14). In this study, the authors used a sample size of n = 3 mice per condition and carried out an orthotopic mammary-fat-pad breast cancer xenograft comparing MCF7 control cells and stable DYRK2 knockdown cells to investigate their ability to produce tumors (14). They found that DYRK2 knockdown cells clearly produce bigger tumors. Furthermore, DYRK2 knockdown cells showed higher invasion potential *in vivo* in an intracardiac injection model (n = 6 mice per condition). The same shRNA DYRK2 depleted cells were used in other studies as well to report the various tumor-suppressor roles of DYRK2 (102, 106). From the study with 715 samples of patients with breast cancer, no correlation was observed between DYRK2 expression and poor outcome in any of the receptor-positive breast cancer subtypes (11). However, TCGA data suggest that mRNA expression of DYRK2 is higher in breast invasive carcinoma and that higher DYRK2 expression correlates with poor survival in overall patients with breast cancer (8, 10, 26). Because mRNA and protein levels sometimes do not correlate, larger analysis looking at DYRK2 protein levels are needed to reach a finite conclusion.

The best way forward is to generate a conditional *lox-cre* mouse model for DYRK2 and generate hemizygous/homozygous deletion of DYRK2 in different subtypes of breast cancer genetically engineered mouse models (GEMMs) (107). Comparative tumor growth in the DYRK2 null *versus* parental GEMM over different subtypes would be a good way of addressing the pending questions on role of DYRK2 in breast cancer.

## DYRK2 in lung cancer: unresolved issues

In 2003, the chromosome 12 region 12q13-14 was found to be amplified in adenocarcinomas of the lung and esophagus, and one of the resident genes, DYRK2, was significantly overexpressed in tumor samples as compared with normal tissues (33). In fact, DYRK2 exhibited the highest mRNA overexpression and highest copy numbers in tumors compared with normal tissue and other genes located in the 12q13-14 chromosomal region, suggesting that the overexpression of DYRK2 is the driving force behind the amplicon (33). This is reiterated in the TCGA lung adenocarcinoma and esophageal cancer cohort wherein tumor samples expressed higher DYRK2 mRNA than normal tissue (8). However, two independent studies report that higher protein or mRNA expression of DYRK2 is a favorable marker in pulmonary adenocarcinoma (66) and non–small-cell lung cancer (NSCLC) (67). In fact, pulmonary adenocarcinoma patients with higher DYRK2 expression exhibited a substantially higher 5-year survival than the group with lower DYRK2 expression. The higher DYRK2 levels associating with negative lymphatic invasion (66). Although the response rates to chemotherapy between the DYRK2-positive and DYRK2-negative patients were not different, patients with DYRK2+ tumors in recurrent NSCLC were suggested to have better outcome with chemotherapy (67). Mechanistically, in lung adenocarcinoma and squamous-cell lung cancer, E3 ubiquitin ligase SIAH2 targets DYRK2 for proteasomal degradation (86). SIAH2 protein and mRNA levels were found to be higher in samples of patients with lung cancer and exhibited a negative correlation with DYRK2 expression (86). Overall, the exact role of DYRK2 in lung neoplasia is still up for debate. Hence, using a similar strategy as suggested previously to generate conditional DYRK2 depletion in genetically engineered lung cancer mouse models for NSCLC, squamous-cell lung cancer, and other subtypes (108) could provide more clarity to this debate.

As reported previously, various global unbiased studies in various cancers have reported DYRK2 as a potential cancer driver with increased copy numbers, overexpression, and higher activity (32, 33, 34). On a similar note, a study using integrated high-resolution microarray analysis of gene copy number and expression in head and neck squamous-cell carcinoma cells reported that DYRK2 had the highest copy number and clear overexpression when compared with other genes in the 12q chromosomal amplicon (109). Furthermore, transcriptomics of blood identified DYRK2 as 1 of 10 potential prognostic biomarkers elevated in high-grade precancerous cervical lesions (110). Thus, unbiased identification of DYRK2 as a protein/kinase involved in potential protumorigenic role along with its substrates such as p53, c-Myc, and c-Jun further fuels the need to stratify cancers into subtypes before embarking on DYRK2 molecular studies. This duality of protumorigenic and antitumorigenic roles has been reported for the paralogue DYRK1A as well (111, 112) (Fig. 3 and Table 1), and hence, there is a clear precedence for such controversial roles in the DYRK family. One way of deconvoluting cancer-type and cell-type functions of a controversial kinase is by generating further tools such as potent and specific small-molecule kinase inhibitors.

## Small-molecule inhibitors of DYRK2

Over the past three decades, various studies have been carried out to identify small-molecule inhibitors of kinases leading to the development of worldwide clinical trials and highly successful therapeutic targets and treatment options (113, 114). For the DYRKs, more than 60 reported small-molecule inhibitors have been published or are available in the public domain. ChEMBL (https://www.ebi.ac.uk/chembl) predicts that there are >1500 potential small molecules that can bind and possibly inhibit DYRK2, including established anticancer drugs sunitinib, erlotinib, afatinib, ruxolitinib, and crizotinib. A significant effort has been focused on development of DYRK1A small-molecule inhibitors because DYRK1A has established roles in neurodegenerative disorders. Consequently, early on the only available DYRK2 inhibitors were those targeting DYRK1A with off-target activity on DYRK2. DYRKs are canonical CMGC kinases and broad-spectrum ATP-competitive kinase inhibitors such as staurosporine and its derivatives inhibit DYRK2 at low nanomolar concentrations (https://www.kinase-screen.mrc.ac.uk/kinase-inhibitors). Although there is a high degree of conservation between the kinase domains of class I and class II DYRKs, structural studies indicated that subtle amino acid substitutions in the hydrophobic inhibitor–docking pocket between DYRK1A and DYRK2 could confer significant degrees of inhibitor specificity (2). Interestingly, these amino acid substitutions contributed to the development and identification of various class-specific and often isoform-specific inhibitors for the DYRKs. Indeed, compound 5j that exhibited more than 100-fold sensitivity for DYRK1A over DYRK1B has no activity for class 2 DYRKs (115). Cocrystallization studies revealed that specific isoleucine to valine replacements in the docking site of curcumin resulted in a larger pocket in the class I DYRKs and thus reduced the shape complementarity to the inhibitor (25). Similarly, ID-8 an indole derivative exhibits an IC_50_ of <100 nM for class 1 DYRKs but >10 μM for class 2 DYRKs, suggesting significant room for developing specific inhibitors for the kinases (116). Similarly, β-carboline derivatives such as harmine or AnnH75 exhibit more *in vivo* and *in vitro* potency for class I than class II DYRKs (Table 2). However, the benzimidazole derivatives such as INDY, TG003, and DYR219 exhibit a pan-DYRK activity *in vitro* and *in vivo* (Table 2) and have been reported to trigger degradation of DYRK proteins when treated in cells (117, 118). This might explain some of the pronounced *in vivo* efficacy compared with *in vitro* observations for DYRK inhibitors wherein prolonged treatment leads to inhibition + degradation of the DYRK target, leading to a significant phenotype. Some promiscuous casein kinase inhibitors derived from benzimidazole potently inhibited DYRK1A and DYRK2 *in vitro* (119, 120). Silmitasertib (CX-4945), a potent and selective inhibitor of CK2 (with IC_50_ of 1 nM *in vitro*), is an orally bioavailable drug currently in phase 1/2 of clinical trials for cancer (121). Intriguingly, silmitasertib potently inhibits both class I and II DYRKs (122). The group did not report the IC_50_ for DYRK2; however, DYRK3 IC_50_ was reported to be 18 nM (122). Because the kinase domains of DYRK2 and DYRK3 are >90% similar at the amino acid level, there is a good chance that silmitasertib could indeed be a potent DYRK2 inhibitor as well. Silmitasertib exhibits blood–brain barrier penetrance similar to brain-penetrant DYR219 (118) and SM07883 (123) and could therefore potentially pharmacologically target the DYRKs in the brain.

**Table 2:** The published DYRK2 inhibitors currently available to the scientific community

Compound	Structure	%Inhibition/IC_50_	Other kinase targets	References
1. Established potent and selective cell-permeable DYRK2 inhibitors				
LDN192960		13 nM	**Haspin (10 nM)**; PIM3 (10 nM) PIM1/2; DYRK1A (122 nM) DYRK1B; DYRK3 (<3 nM)	(26, 124, 125)
GSK626616		<1 nM	**DYRK3 (0.7 nM)**; DYRK1A/B; CK	(131)
Leucettine L41		35 nM	**CLK1 (15 nM)** CLK 2 DYRK1A (40 nM) GSK3 (410 nM)	(132)
EHT 5372		10.8 nM	**DYRK1A (0.22 nM)** DYRK1B (0.28 nM); CLK1 (22.8 nM) CLK2 (88.8 nM); DYRK3 (93.2 nM); GSK3alpha (7.44 nM) GSK3Beta (221 nM)	(133)
EHT 1610		3.16 nM	**DYRK1A (0.36 nM)** DYRK1B (0.59 nM) CLK1 (11 nM) CLK2 (32.5 nM) CLK3 (1420 nM) DYRK3 (21.1 nM) GSK3 (9.11 nM)	(134)
TG003		80% inhibition at 1000 nM	DYRK1A (24.01 nM) DYRK1B (34.39 nM) **CLK1/2/3** DYRK3	https://www.kinase-screen.mrc.ac.uk/kinase-inhibitors (133)
INDY		27.7 nM	**DYRK1A (139 nM)** DYRK1B (69.2 nM) CLK1/2/3; DYRK3; CK	(135, 136)
2. Brain-penetrant DYRK2 inhibitors				
DYR219	benzimidazole analog	90% inhibition at 10,000 nM	**DYRK1A (34 nM)** DYRK1B (29 nM) GSK3; CDK5	(118)
SM07883	3-Acylamino-isoquinoline analog	16 nM	**DYRK1A (1.6 nM)** DYRK1B (8 nM) GSK3Beta (10.8 nM) CLK4 (3 nM)	(123)
3. Potent DYRK2 lead compounds/analogs reported				
RD0392		45.6 nM	**DYRK1A (60.2 nM)** DYRK1B (53.3 nM)	(136)
Abbott COT/TPL2 compound 41		70 nM	**COT/TPL2**; DYRK3; DYRK1A	https://www.kinase-screen.mrc.ac.uk/kinase-inhibitors
Fluoro-DANDY analog 5 g		<50 nM	**DYRK1A (9.34 nM)** **DYRK1B**; DYRK3 CDK2/5 GSK3 ERK2	(137)
SC97202		13.8 nM	**DYRK1A (<0.1 nM)** **DYRK1B (0.31 nM)** DYRK3 (23.38 nM)	(138)
LY2835219		61 nM	**CDKs** PIM1 (50 nM) HIPK2 (31 nM) CK2 (117 nM) GSK3β (192 nM) JNK (389 nM)	(139)
Milciclib		48 nM	**CDK2 (45 nM)** CLK1 (3 nM) CLK2 (1 nM) CLK4 (13 nM) DYRK1A (17 nM) DYRK1B (5 nM)	(140)
PAC1		140 nM	**CLK1 (430 nM)** **CLK4 (400 nM)** **DYRK1A (230 nM)** **DYRK1B (400 nM)** **DYRK3 (280 nM)**	(141)
Compound 6i		130 nM	**DYRK1A (210 nM)**	(142)
4. Other identified DYRK2 inhibitors				
Curcumin		5 nM	Highly nonspecific at >15-μM concentrations	(25)
Harmine		900 nM	**CLKs; DYRK1A (80 nM)** **DYRK3 (800 nM)**; monoamine oxidase	(127)
Ro3306		80% inhibition at 1000 nM	**CDKs**; PKC; SGK	https://www.kinase-screen.mrc.ac.uk/kinase-inhibitors
BX-795		72% inhibition at 1000 nM	**PDK1**; AMPK; NUAK1	https://www.kinase-screen.mrc.ac.uk/kinase-inhibitors (143)
A-443654		74% inhibition at 100 nM	**AKT**; PKA; DYRK1A; DYRK3	https://www.kinase-screen.mrc.ac.uk/kinase-inhibitors
AnnH75		58% inhibition at 1000 nM	**CLK1; DYRK1A (181 nM)** Haspin	(144)
WNK-463		80% inhibition at 1000 nM	**WNKs**	https://www.kinase-screen.mrc.ac.uk/kinase-inhibitors
GNF2133		95% inhibition at 2000 nM	**DYRK1A (6.2 nM)** **DYRK1B**; DYRK3; CLK1/2; RIPK2; FLT3/4	(145)

The kinase in bold format indicates the original target reported for the compound. IC_50_ values are provided where available.

A recently well-characterized DYRK2 inhibitor is the acridine analog, LDN192960 (26). Initially developed as a Haspin inhibitor (124, 125), LDN192960 inhibited DYRK2 with 13 nM IC_50_ and exhibited antitumor activity in mouse models of MM and TNBC (26). Interestingly, LDN192960 exhibited a ‘mixed’ mode of DYRK2 inhibition and cocrystal structure of LDN192960 with DYRK2 revealed that two water molecules mediated multiple hydrogen bonds between LDN192960 and DYRK2 active pocket (26). Similar water molecule–mediated interaction was also observed in the cocrystal structure of DYRK1A with inhibitor DJM2005 wherein a water molecule facilitated hydrogen bonding to further stabilize the inhibitor bound structure (2). LDN192960 reduced tumor burden of syngrafted and patient-derived xenografted mouse models of TNBC and delayed bone degeneration of allografted MM mouse models (26). In cells, LDN192960 exhibited potent cytotoxicity toward cancer lines with minimal impact on noncancerous cells (26). In fact, LDN192960 induced cytotoxicity to CD138^+^ primary myeloma cells of patients with significantly less impact on matched peripheral mononuclear cells (26). LDN192960 also exhibited additive effects in combination with FDA-approved proteasome inhibitor carfilzomib in inducing cytotoxicity in myeloma cells (26). LDN192960 was bioavailable *in vivo* and a dose of 50 mg/Kg body weight was sufficient to target neoplasia (26). Thus, LDN192960 inhibited DYRK2 *in vivo* and reduced 26S proteasome activity and thereby targeted proteasome-dependent neoplastic diseases such as TNBC and MM (25, 26).

Interestingly, curcumin inhibits DYRK2 with an IC_50_ of 5 nM and cocrystal structure revealed that curcumin binds to the active site pocket of DYRK2 *via* hydrophobic interactions (25). Curcumin is highly promiscuous, nonbioavailable, and unstable in the serum-free solution and has been labeled as both a pan-assay interference compound and an invalid metabolic panacea (126). Curcumin aggregates at concentrations greater than 15 μM, and most studies reporting controversial biological targets for curcumin used high 20- to 100μM concentrations leading to possible false positives (126). However, at lower 1- to 3μM concentrations, curcumin ablates DYRK2-mediated 26S proteasome phosphorylation in cells, reduces proteasome activity, and impairs cell proliferation in TNBC and MM cell lines *in vitro* and *in vivo* (25). DYRK2 KO cells exhibit no further off-target effects on proteasome activity with curcumin (25). Although neither a viable drug scaffold nor a highly potent kinase inhibitor, curcumin could serve as a decent compound for DYRK2 inhibition when used with proper controls.

In Table 2, we have listed those published inhibitors which have been tested directly on DYRK2 activity *in vivo* or *in vitro* along with a few interesting scaffolds identified in medicinal chemistry publications (https://www.kinase-screen.mrc.ac.uk/kinase-inhibitors) (127, 128). An important observation is that not a single one of these DYRK2 inhibitors exhibit protumorigenic/pro–cell-proliferation properties.

## Conclusion and future perspectives

The controversial role of DYRK2 in cancer is evident (Fig. 3). Recent review articles mentioned the conflicting literature on DYRK2 (8, 28) and reported that mRNA expression data show that DYRK2 levels are higher in invasive breast carcinoma and lung adenocarcinoma than normal/adjacent tissue control (8, 26). However, others maintain that DYRK2 is a major tumor suppressor across all breast cancer subtypes and that DYRK2 depletion promotes proliferation (27). Indeed, being a CMGC kinase with a +1Pro active site, there are expected redundancies between DYRK2, other DYRKs, and possibly other CMGC kinases. A recent review article provides a list of substrates exhibiting overlapping DYRK kinases phosphorylating the same residues (8). Furthermore, as a member of the EDVP complex, cancer-specific mutations can alter DYRK2 substrate signature in specific cancer cells, which could be either tumor suppressive (3) or oncogenic (89). Although immunohistochemistry of tumors in patients with glioma shows lower levels of DYRK2 correlates with poorer survival (65), a receptor tyrosine kinase–transduced p53 null glioma mouse model exhibits higher DYRK2 activity and potentially altered signaling with diverse substrates (34). It is a definite possibility that we have just seen the tip of the iceberg when it comes to deconvoluting the redundancy and substrate overlap between the DYRKs and other CMGC kinases. As already shown, meticulous inhibitor screens and development of phospho-specific antibodies to the substrates could pave the way to dissect specificities and potential redundancies between the CMGC kinases (80). With the establishment of genetic engineering CRISPR KO/knock-in strategies and advanced quantitative phosphoproteomics, we might be able to dig deeper into identifying novel substrates and mechanisms in various cancer types. One major way forward would be to generate a conditional KO mouse model of DYRK2 in various cancer GEMM backgrounds to study tumor development and thereby ascertain the specific role of DYRK2 in each cancer subtype. A latest study reports that mouse embryonic fibroblasts with DYRK2 deletion exhibits significant downregulation of major cell cycle and proliferation drivers and markers such as Ki67, Aurora kinase A, PLK1, Bub1, and Bub1b (19). Although not in a cancer model, these observations are consistent with those observed in TNBC tumors where depletion of DYRK2 leads to reduced cell proliferation with greatly reduced Ki67 (10, 25, 26). However, DYRK2 could drive tumor-suppressor functions pairing with substrates reported in Figure 3 and Table 1 or other yet-undiscovered substrates in cancer type–specific mechanisms (27). In future, the roles of DYRK2 in cancer need to be approached in a more holistic way using multiple controlled models in each study including but not limited to genetic depletions and biochemical analyses, using specific inhibitors, *in vivo* animal models, and *in vitro* cell-based assays. Although overexpression of mRNA in cancers does indicate a potential oncogenic role, correlating that to corresponding increase of protein levels is important because mRNA and protein level often do not correlate in tumor samples. Before immunohistochemistry analysis on patient samples, proper antibody optimization steps are necessary while data analyses and sample size determinations need to be supported by proper statistical principles. Furthermore, ectopic overexpression of DYRK2 often results in false-positive substrate phosphorylation/binding and such experiments should always be accompanied with controls to ascertain the physiological/bona fide roles of the kinase. Like its paralogue DYRK1A (111, 112) and many other kinases, DYRK2 may indeed play both protumorigenic and antitumorigenic roles in different cancer types and subtypes, which is often determined by spatiotemporal interactions between kinases and specific substrates. Novel cancer therapeutic targets are a need of the hour, and hence, controversies delaying the establishment of a potential target or a tumor suppressor need to be objectively and quickly addressed. Deconvolution of the enigmatic roles of DYRK2 in various cancer types and subtypes should be prioritized among those in the field making our tools and expertise available for the greater scientific community in this endeavor.

## Conflict of interest

The authors declare that they have no conflicts of interest with the contents of this article.
